# Usefulness of baseline statin therapy in non-obstructive coronary artery disease by coronary computed tomographic angiography: From the CONFIRM (COronary CT Angiography EvaluatioN For Clinical Outcomes: An InteRnational Multicenter) study

**DOI:** 10.1371/journal.pone.0207194

**Published:** 2018-12-12

**Authors:** Yun-Kyeong Cho, Chang-Wook Nam, Bon-Kwon Koo, Joshua Schulman-Marcus, Bríain Ó. Hartaigh, Heidi Gransar, Yao Lu, Stephan Achenbach, Mouaz Al-Mallah, Daniele Andreini, Jeroen J. Bax, Matthew J. Budoff, Filippo Cademartiri, Tracy Q. Callister, Hyuk-Jae Chang, Kavitha Chinnaiyan, Benjamin J. W. Chow, Ricardo C. Cury, Augustin Delago, Gudrun Feuchtner, Martin Hadamitzky, Jörg Hausleiter, Philipp A. Kaufmann, Yong-Jin Kim, Jonathon Leipsic, Erica Maffei, Hugo Marques, Gianluca Pontone, Gilbert L. Raff, Ronen Rubinshtein, Leslee J. Shaw, Todd C. Villines, Daniel S. Berman, Erica C. Jones, Jessica M. Peña, Fay Y. Lin, James K. Min

**Affiliations:** 1 Department of Cardiology, Keimyung University Dongsan Medical Center, Daegu, Korea; 2 Department of Internal Medicine and Cardiovascular Center, Seoul National University College of Medicine, Seoul, Korea; 3 Department of Radiology, NewYork-Presbyterian Hospital and the Weill Cornell Medical College, New York, New York, United States of America; 4 Department of Imaging, Cedars-Sinai Heart Institute, Cedars-Sinai Medical Center, Los Angeles, California, United States of America; 5 Department of Healthcare Policy and Research, New York-Presbyterian Hospital and the Weill Cornell Medical College, New York, New York, United States of America; 6 Department of Cardiology, Friedrich-Alexander-University Erlangen-Nuremburg, Germany; 7 King Saud bin Abdulaziz University for Health Sciences, King Abdullah International Medical Research Center, King AbdulAziz Cardiac Center, Ministry of National Guard, Health Affairs, Riyadh, Saudi Arabia; 8 Centro Cardiologico Monzino, IRCCS, Milan, Italy; 9 Department of Cardiology, Leiden University Medical Center, Leiden, The Netherlands; 10 Department of Medicine, Los Angeles Biomedical Research Institute, Torrance, California, United States of America; 11 Cardiovascular Imaging Center, SDN IRCCS, Naples, Italy; 12 Tennessee Heart and Vascular Institute, Hendersonville, Tennessee, United States of America; 13 Division of Cardiology, Severance Cardiovascular Hospital and Severance Biomedical Science Institute, Yonsei University College of Medicine, Yonsei University Health System, Seoul, South Korea; 14 Division of Cardiology, William Beaumont Hospital, Royal Oak, Michigan, United States of America; 15 Department of Medicine and Radiology, University of Ottawa, Ontario, Canada; 16 Department of Radiology, Miami Cardiac and Vascular Institute, Miami, Florida, United States of America; 17 Capitol Cardiology Associates, Albany, New York, United States of America; 18 Department of Radiology, Medical University of Innsbruck, Innsbruck, Austria; 19 Department of Radiology and Nuclear Medicine, German Heart Center Munich, Munich, Germany; 20 Medizinische Klinik I der Ludwig-Maximilians-Universität München, Munich, Germany; 21 Department of Nuclear Medicine, University Hospital Zurich, Zurich, Switzerland; 22 Department of Medicine and Radiology, University of British Columbia, Vancouver, British Columbia, Canada; 23 Department of Radiology, Area Vasta 1/ASUR Marche, Urbino, Italy; 24 UNICA, Unit of Cardiovascular Imaging, Hospital da Luz, Lisboa, Portugal; 25 Department of Cardiology at the Lady Davis Carmel Medical Center, The Ruth and Bruce Rappaport School of Medicine, Technion-Israel Institute of Technology, Haifa, Israel; 26 Cardiology Service, Walter Reed National Military Center, Bethesda, Maryland, United States of America; 27 Department of Imaging and Medicine, Cedars Sinai Medical Center, Los Angeles, California, United States of America; University of Palermo, ITALY

## Abstract

**Background:**

The extent to which the presence and extent of subclinical atherosclerosis by coronary computed tomography angiography influences a potential mortality benefit of statin is unknown. We evaluated the relationship between statin therapy, mortality, and subclinical atherosclerosis.

**Methods:**

In the CONFIRM study, patients with normal or non-obstructive plaque (<50% diameter stenosis) for whom data on baseline statin use was available were included. Coronary artery calcium (CAC) was quantified using the Agatston score. The extent of non-obstructive coronary atherosclerosis was quantified using the segment involvement score (SIS). 8,016 patients were followed for a median of 2.5 years with analysis of all-cause mortality and major adverse cardiac events (MACE) including all-cause mortality, myocardial infarction, unstable angina, target vessel revascularization, and coronary artery disease-related hospitalization.

**Results:**

1.2% of patients experienced all-cause mortality. Patients not on baseline statin therapy had a stepwise increased risk of all-cause mortality by CAC (relative to CAC = 0; CAC 1–99: hazard ratio [HR] 1.65, CAC 100–299: HR 2.19, and CAC≥300: HR 2.98) or SIS (relative to SIS = 0; SIS 1: HR 1.62, SIS 2–3: 2.48 and SIS≥4: 2.95). Conversely, in patients on baseline statin therapy, there was no significant increase in mortality risk with increasing CAC (p value for interaction = 0.049) or SIS (p value for interaction = 0.007). The incidence of MACE was 2.1%. Similar to the all-cause mortality, the risk of MACE was increased with CAC or SIS strata in patient not on baseline statin therapy. However, this relation was not observed in patient on baseline statin therapy.

**Conclusion:**

In individuals with non-obstructive coronary artery disease, increased risk of adverse events occurs with increasing CAC or SIS who are not on baseline statin therapy. Statin therapy is associated with a mitigation of risk of cardiac events in the presence of increasing atherosclerosis, with no particular threshold of disease burden.

## Introduction

Coronary artery calcium (CAC) scoring is a robust method for risk prediction of major adverse cardiac events (MACE), and current societal guidelines recommend a threshold of ≥300 Agatston units for consideration of statin treatment [1]. Coronary computed tomographic angiography (CCTA) is a contrast-enhanced anatomic imaging method that permits direct visualization of both calcified and non-calcified atherosclerotic plaque that also allows for effective prognostication of risk of MACE [2–7]. In patients with non-obstructive coronary artery disease (CAD) by CCTA, the comparative effect of statin therapy on MACE for individuals with evident CAC or atherosclerotic plaque by CCTA is unknown. Thus, in a prospective multinational cohort of individuals undergoing CAC and CCTA without obstructive CAD, we sought to identify whether there was a threshold of CAC- and CCTA-identified CAD wherein statin therapy was associated with reduced mortality risk.

## Materials and methods

### Study population

**Fig 1:**
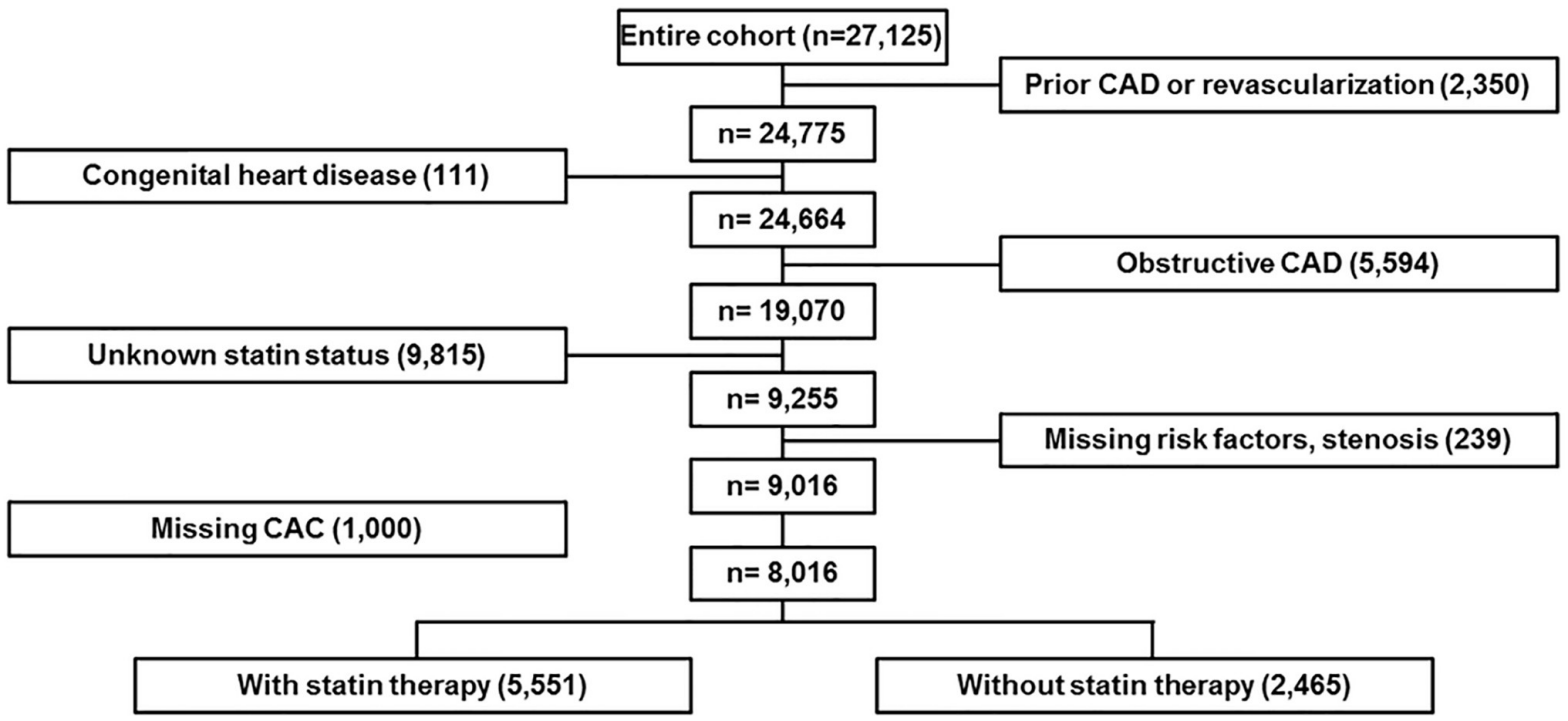
Flow diagram for patient enrollment. A total of 8,016 patients met the inclusion criteria.

Details of the CONFIRM (COronary CT Angiography EvaluatioN For Clinical Outcomes: An InteRnational Multicenter) study have been described elsewhere [8]. In brief, 27,125 consecutive patients enrolled in this global multicenter cardiac CT registry underwent coronary CCTA at 12 cluster sites in 6 countries (Canada, Germany, Italy, Korea, Switzerland, United States) between February 2003 and December 2009. Patients with a history of myocardial infarction or coronary revascularization (coronary artery bypass and/or percutaneous coronary intervention) (n = 2,350), or congenital heart disease (n = 111) were excluded from analysis. A further 16,648 patients were excluded including those with obstructive CAD (≥50% luminal diameter stenosis) as diagnosed by CCTA (n = 5,594), and those with missing data regarding the use of statins (n = 9,815), risk factors (n = 239), or CAC (n = 1000) were also excluded. A total of 8,016 patients met the inclusion criteria and comprised the study sample (Fig 1). The Institutional Review Board of Weil Cornell Medical College approved the study and its procedures, including coordination with other ethics committees. Each of the contributing centers received ethics approval from their respective institutional review boards, and written informed consent was obtained from the study participants.

### Study variables

At the time of CCTA examination, patient’s information was prospectively collected and recorded in site-specific case report forms (CRFs). Patients treated for or with a prior diagnosis of hypertension, diabetes, or dyslipidemia, a family history of premature CAD or a history of smoking were categorized as having that cardiovascular risk factor. Specifically, systemic arterial hypertension was defined as a documented history of high blood pressure or treatment with antihypertensive medication. Diabetes mellitus was defined as diagnosis of diabetes confirmed previously by a physician and/or use of insulin or oral hypoglycemic agents. Dyslipidemia was defined as known but untreated dyslipidemia or current treatment with lipid-lowering medications. A family history of premature CAD was defined as a primary relative with a diagnosis early in life (i.e., mother <65 years of age or father <55 years of age). A positive smoking history was defined as current smoking or cessation of smoking within 3 months of examination. Self-reported use of statin medication was evaluated at the time of enrolment.

### Definition of CCTA measures

Image data were acquired by CT scanners of ≥64-detector rows. Patient preparation, acquisition, and interpretation of CCTA and CAC score data were performed in accordance with the Society of Cardiovascular Computed Tomography Guidelines [9]. For the present analysis, coronary stenoses were defined as none (0% stenosis without plaque) and non-obstructive (1–49% diameter stenosis) CAD. The CAC score was determined based on the scoring system described by Agatston et al. [10]. The CAC score was categorized into 4 strata as: 0, 1–99, 100–299, and ≥300 according to current guidelines [11]. The extent of atherosclerotic burden was determined by a segment-involvement score (SIS) based on a 16-segment coronary model, which reflects the number of coronary segments possessing atherosclerotic plaque (minimum = 0; maximum = 16) [12]. SIS was also categorized as 0, 1, 2–3, and ≥4 segments in the current study population.

### Patient follow-up

Primary endpoint was all-cause mortality and secondary endpoint was major adverse cardiac events (MACE). According to the study protocol, MACE was defined as all-cause mortality, myocardial infarction, unstable angina, target vessel revascularization, and CAD-related hospitalization. Event data were ascertained at each local institution by direct patient query, through medical records at non-US sites or national all-cause mortality records at US sites. Data coordinating center and independent biostatistician checked the database to enhance data quality, and they only knew the participants only by study identifier number.

### Statistical methods

Continuous variables are presented as means with standard deviations, and categorical variables as counts with proportions. Between-group differences according to statin use were compared by use of a Wilcoxon rank-sum test for continuous variables, and chi-square test for categorical variables. Unadjusted comparisons of the primary outcome according to the presence and magnitude of the CAC and SIS scores stratified by statin therapy were performed using Kaplan-Meier survival curves with log-rank tests. A multivariable Cox proportional regression model reporting hazard ratios with 95% confidence intervals (95% CI) was employed to examine differences in the risk of all-cause mortality and MACE according to statin therapy, while adjusting for age, gender, and traditional cardiovascular risk factors such as hypertension, diabetes mellitus, dyslipidemia, family history and current smoking. All statistical analyses were performed using SPSS (version 19.0.0, IBM, New York). A two-tailed p value <0.05 was considered statistically significant.

## Results

### Study population

Of 8,016 patients, the incidence of all-cause mortality was 1.2% (99 events) and that of MACE was 2.1% (165 events) during a median follow-up of 2.5 years. Baseline characteristics of the study cohort are presented in Tables 1 and 2. Patients on baseline statin therapy tended to be older and have more CAD risk factors (p <0.001 for all) compared with those who were not on statin therapy. Both higher CAC and SIS scores were associated with older age as well as a higher prevalence of hypertension, diabetes mellitus, and dyslipidemia.

**Table 1:** Baseline characteristics.

	Overall	Without statin	With statin	p value
	(N = 8016)	(N = 5551)	(N = 2465)	
Age	57.1±11.8	55.6±12.2	60.7±10.2	<0.001
Male gender	4152 (51.8)	2895 (52.2)	1257 (51.0)	0.338
Hypertension	3930 (49.0)	2513 (45.3)	1417 (57.5)	<0.001
Diabetes mellitus	978 (12.2)	530 (9.5)	448 (18.2)	<0.001
Dyslipidemia	4544 (56.7)	2500 (45.0)	2044 (82.9)	<0.001
Family history	1982 (24.7)	1294 (23.3)	688 (27.9)	<0.001
Current smoking	1277 (15.9)	917 (16.5)	360 (14.6)	0.031
Body mass index	26.3±4.7	26.3±4.8	26.3±4.7	0.688
Renal insufficiency	16 (0.2)	10 (0.2)	6 (0.2)	0.591
Peripheral vascular disease	112 (1.4)	83 (1.5)	29 (1.2)	0.205
Cerebrovascular disease	73 (0.9)	51 (0.9)	22 (0.9)	0.965
Total cholesterol	191.8±43.0	192.4±42.9	190.3±43.1	0.089
LDL cholesterol	118.0±35.8	118.3±36.3	117.3±34.6	0.332
HDL cholesterol	53.6±16.3	53.9±16.6	53.1±15.6	0.066
CAC	65.6±242.1	46.8±198.6	108.1±314.9	<0.001
SIS	1.0±1.7	0.7±1.5	1.5±2.0	<0.001

LDL, low-density lipoprotein; HDL, high-density lipoprotein; CAC, coronary artery calcium; SIS, segment involvement score.

**Table 2:** Baseline characteristics by CAC or SIS categories.

	CAC 0	CAC 1–99	CAC 100–299	CAC ≥300	p value	SIS 0	SIS 1	SIS 2–3	SIS ≥4	p value
	(N = 4858)	(N = 2060)	(N = 623)	(N = 475)		(N = 4969)	(N = 1254)	(N = 1097)	(N = 696)	
Age	53.5±11.5	60.9±9.9	65.1±9.1	68.0±9.4	<0.001	54.0±11.6	60.3±10.4	62.3±9.9	65.6±10.1	<0.001
Male gender	2273 (46.8)	1190 (57.8)	381 (61.2)	308 (64.8)	<0.001	2343 (47.2)	698 (55.7)	668 (60.9)	443 (63.6)	<0.001
Hypertension	2128 (43.8)	1136 (55.1)	366 (58.7)	300 (63.2)	<0.001	2204 (44.4)	665 (53.0)	611 (55.7)	450 (64.7)	<0.001
Diabetes mellitus	423 (8.7)	338 (16.4)	111 (17.8)	106 (22.3)	<0.001	461 (9.3)	173 (13.8)	198 (18.0)	146 (21.0)	<0.001
Dyslipidemia	2561 (52.7)	1277 (62.0)	422 (67.7)	284 (59.8)	<0.001	2620 (52.7)	782 (62.4)	684 (62.4)	458 (65.8)	<0.001
Family history	1176 (24.2)	507 (24.6)	169 (27.1)	130 (27.4)	0.219	1195 (24.0)	290 (23.1)	322 (29.4)	175 (25.1)	0.001
Current smoking	757 (15.6)	327 (15.9)	103 (16.5)	90 (18.9)	0.279	777 (15.6)	202 (16.1)	174 (15.9)	124 (17.8)	0.532
Body mass index	26.4±4.8	26.0±4.5	26.2±5.0	26.2±4.7	0.012	26.4±4.7	26.2±4.9	26.1±4.9	25.9±4.6	0.007
Renal insufficiency	9 (0.2)	5 (0.2)	1 (0.2)	1 (0.2)	0.968	8 (0.2)	3 (0.2)	5 (0.5)	0	0.201
Peripheral vascular disease	70 (1.4)	22 (1.1)	9 (1.4)	11 (2.3)	0.259	66 (1.3)	22 (1.8)	11 (1.0)	13 (1.9)	0.242
Cerebrovascular disease	38 (0.8)	17 (0.8)	8 (1.3)	10 (2.1)	0.187	33 (0.7)	16 (1.3)	12 (1.1)	12 (1.7)	0.201
Total cholesterol	194.5±45.0	187.7±39.8	183.8±37.0	189.6±37.1	<0.001	194.2±45.1	187.3±42.1	187.3±34.5	187.2±36.6	<0.001
LDL cholesterol	119.8±37.3	115.6±33.1	112.2±31.3	116.0±32.5	<0.001	119.8±38.0	114.1±31.7	114.9±29.2	115.6±31.5	<0.001
HDL cholesterol	54.4±16.6	52.2±15.9	52.9±16.4	52.4±13.8	<0.001	54.0±16.4	53.6±16.9	53.0±15.8	52.2±15.1	0.115
CAC	0	29.9±27.4	174.9±56.3	748.1±671.9	<0.001	9.7±123.5	45.4±141.8	121.3±239.9	413.8±538.8	<0.001
SIS	0.2±0.6	1.4±1.4	3.0±2.0	4.5±2.5	<0.001	0	1.0±0.0	2.4±0.5	5.4±1.7	<0.001

LDL, low-density lipoprotein; HDL, high-density lipoprotein; CAC, coronary artery calcium; SIS, segment involvement score.

### CAC and statin therapy

**Fig 2:**
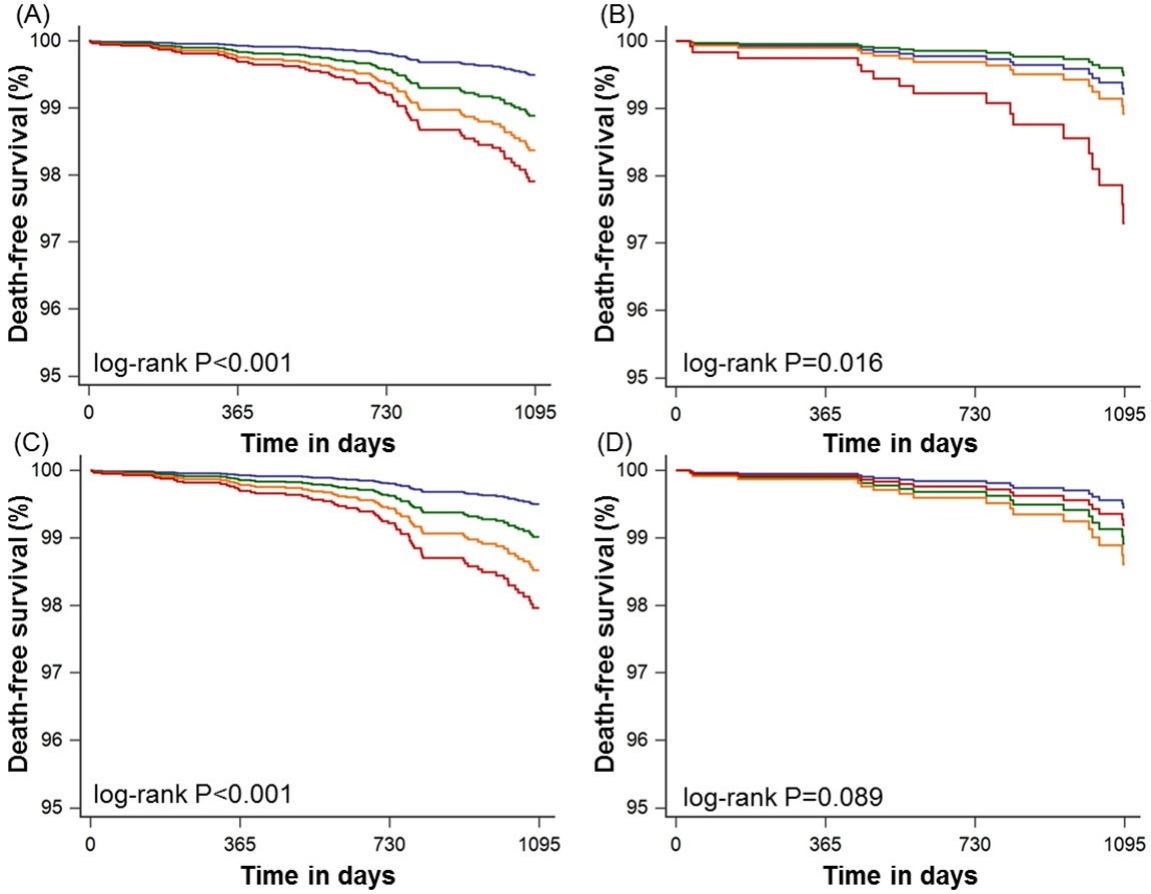
Kaplan-Meier survival curves for all-cause mortality-free survival. (A) coronary artery calcium score categories for patients without statin therapy, (B) coronary artery calcium score categories for patients with statin therapy, (C) segment involvement score categories for patients without statin therapy, and (D) segment involvement score categories for patients with statin therapy. Blue, green, orange, and red lines indicate 0, 1–99, 100–299, and ≥300 coronary artery calcium score categories. Blue, green, orange, and red lines indicate 0, 1, 2–3, and ≥4 segment involvement score categories.

In the overall population, there was a stepwise increased risk of all-cause mortality by strata of CAC after adjustment for covariates (Table 3). This association was influenced by the presence of baseline statin therapy (Fig 2, Table 3). Specifically, the stepwise relationship between increasing CAC and increased mortality was preserved in patients not on baseline statin therapy. However, there was no significant association between individual strata of CAC and all-cause mortality after adjustment for clinical variables, although there was a borderline association of increased risk for CAC≥300 (HR = 3.05, 95% CI 0.92–10.09, p = 0.07). As compared to patients taking statins, patients not on statin therapy had significantly higher risks of all-cause mortality according to increasing CAC strata except for those with CAC ≥300 (p value for interaction = 0.049).

**Table 3:** Adjusted association between all-cause mortality, CAC, SIS, and baseline statin therapy^a^.

	Overall population		Without statin therapy		With statin therapy	
	Hazard ratio (95% CI)	p value	Hazard ratio (95% CI)	p value	Hazard ratio (95% CI)	p value
CAC 0	1.00		1.00		1.00	
CAC 1–99	1.29 (0.76–2.17)	0.346	1.65 (0.92–2.95)	0.097	0.56 (0.17–1.91)	0.357
CAC 100–299	1.90 (1.00–3.61)	0.052	2.19 (1.04–4.65)	0.041	1.19 (0.34–4.19)	0.789
CAC ≥300	2.86 (1.55–5.27)	<0.001	2.98 (1.44–6.16)	0.004	3.05 (0.92–10.09)	0.068
SIS 0	1.00		1.00		1.00	
SIS 1	1.54 (0.85–2.78)	0.154	1.62 (0.81–3.24)	0.171	1.37 (0.44–4.29)	0.586
SIS 2–3	2.25 (1.32–3.83)	0.003	2.48 (1.34–4.61)	0.004	1.62 (0.56–4.71)	0.375
SIS ≥4	2.07 (1.13–3.77)	0.019	2.95 (1.50–5.81)	0.002	0.84 (0.21–3.33)	0.800

CAC, coronary artery calcium; SIS, segment involvement score.

^a^Adjusted for age, male gender, hypertension, diabetes mellitus, dyslipidemia, family history and current smoking.

Although the risk of MACE was increased by strata of CAC in the overall population, this finding was attenuated according to the baseline statin therapy (Table 4).

**Table 4:** Adjusted association between major adverse cardiac events (MACE)^a^, CAC, SIS, and baseline statin therapy^b^.

	Overall population		Without statin therapy		With statin therapy	
	Hazard ratio (95% CI)	p value	Hazard ratio (95% CI)	p value	Hazard ratio (95% CI)	p value
CAC 0	1.00		1.00		1.00	
CAC 1–99	1.62 (1.07–2.45)	0.022	2.15 (1.30–3.56)	0.003	0.76 (0.36–1.59)	0.462
CAC 100–299	2.64 (1.60–4.37)	<0.001	2.54 (1.28–5.01)	0.007	2.16 (1.03–4.55)	0.042
CAC ≥300	4.63 (2.87–7.45)	<0.001	4.91 (2.65–9.11)	<0.001	3.84 (1.81–8.13)	<0.001
SIS 0	1.00		1.00		1.00	
SIS 1	2.22 (1.43–3.47)	<0.001	1.82 (1.02–3.24)	0.043	2.65 (1.30–5.43)	0.008
SIS 2–3	2.84 (1.85–4.37)	<0.001	3.13 (1.86–5.27)	<0.001	1.95 (0.91–4.18)	0.088
SIS ≥4	3.48 (2.18–5.55)	<0.001	3.19 (1.75–5.82)	<0.001	3.54 (1.67–7.48)	0.001

CAC, coronary artery calcium; SIS, segment involvement score.

^a^MACE was defined as a composite of all-cause mortality, myocardial infarction, unstable angina, target vessel revascularization, and coronary artery disease-related hospitalization

^b^Adjusted for age, male gender, hypertension, diabetes mellitus, dyslipidemia, family history and current smoking.

### SIS and statin therapy

In the overall population, there was a stepwise increased risk of all-cause mortality by increasing strata of SIS after adjustment of covariates (Table 3). Compared to patients with SIS = 0, patients with SIS >1 was associated with higher risk of all-cause mortality (p <0.05). This association was influenced by the presence of baseline statin therapy (Fig 2, Table 3). Relative to those with SIS = 0 not taking baseline statins, the adjusted risk of all-cause mortality increased stepwise with increasing SIS, with significantly increased mortality hazard for SIS 2–3 and SIS ≥4. Conversely, this stepwise association was attenuated by the presence of baseline statin therapy (p value for interaction = 0.007). An increased mortality hazard was not observed in patients with any degree of SIS on baseline statin therapy.

A stepwise increased risk of MACE by increasing strata of SIS was also observed in the overall population (Table 4). Compared to patients with SIS = 0, patients with SIS ≥1 was associated with higher risk of MACE (p <0.05). Relative to those with SIS = 0 not taking baseline statins, the adjusted risk of MACE was significantly increased in patients with SIS ≥1. However, this association was abated in patients with statin therapy.

## Discussion

In this prospective multinational cohort study of individuals undergoing CCTA, we identified a stepwise increased risk of all-cause mortality for individuals with increasing atherosclerotic burden despite the absence of anatomically obstructive CAD. Importantly, this association was modified by the presence or absence of baseline statin therapy. In contrast to individuals not on baseline statin therapy, the baseline use of statins was associated with a mitigation of increased mortality risk despite the presence or burden of non-obstructive atherosclerosis. These findings support the notion that statins may be beneficial in even patients with lower degrees of subclinical atherosclerosis as detected by CCTA.

Prior evidence has demonstrated that a substantial proportion of cardiac events arise from non-obstructive coronary stenoses [13, 14]. For instance, the PROSPECT (Providing Regional Observations to Study Predictors of Events in the Coronary Tree) study observed that many cardiac events arise from angiographic non-culprit lesions, especially those with high plaque burden and thin-cap fibroatheroma detected by gray-scale intravascular ultrasound [15]. Longitudinal data has further demonstrated an increased event risk in patients with non-obstructive CAD as detected by invasive angiography or CCTA [16, 17]. Accordingly, the present investigation of patients with non-obstructive CAD observed an incremental occurrence of mortality according to increasing CAC or SIS in patients not on statin therapy. These findings are clinically significant, in that most conventional testing (e.g. stress testing) for CAD may not detect non-obstructive atherosclerosis.

In the past decade, numerous randomized controlled trials have documented that statin therapy not only prevents cardiovascular events but also Finimproves survival in patients with varying degrees of clinical CAD, although these trials did not specifically study patients with anatomically non-obstructive CAD [18–21]. Statins are the leading candidate for pharmacologic prevention in such patients based on the observation that statins could slow disease progression and elicit stabilization of vulnerable plaque [22, 23]. Hoffmann et al. [24] evaluated the influence of statins on plaque progression by volumetric measurement using CCTA, and found that statins significantly slowed the growth of non-calcified plaques after adjusting for LDL-C levels and cardiac risk factors. It can be argued that the importance of statin therapy in non-significant stenosis was demonstrated in the FAME 2 (Fractional Flow Reserve versus Angiography for Multivessel Evaluation 2) study, in which patients who had been deferred with functionally non-significant lesions were treated to the target of LDL-C <70 mg/dl with clinical outcomes as favorable as those associated with revascularization [25]. As well as plaque stabilization and regression, statins act beneficially through different pleiotropic effects on inflammation, fibrosis, endothelial function, thrombosis, and coagulation. Via these mechanisms, statin might also contribute to the reduction in all-cause mortality.

In light of this evidence, recent guidelines suggest that high CAC (i.e., greater than 300 Agatston units) should be taken into incorporated into statin decision-making in certain primary prevention settings [1]. The results of the present study suggest potential benefit for patients with even lower degrees of CAC or non-obstructive CAD by CCTA [26]. Importantly, we identified no lower limit of CAC wherein statin therapy did not mitigate mortality risk. These findings suggest that a binary cutoff of CAC≥300 for guiding statin treatment may not be warranted, as those with lower scores appear to also derive significant risk attenuation. Furthermore, this attenuation of risk was also observed in patients with SIS>1 on baseline statins, which extends the hypothesis that any degree of atherosclerosis by CCTA should factor into statin decision-making.

In the use of statin for the primary prevention of cardiovascular disease, there is still the issue of gender-dependent efficacy. Recent meta-analysis including 174,000 patients showed the effect of statin therapy on mortality and cardiac events were comparable among women and men, matched for cardiovascular risk [27]. When we analyzed the association between adverse events and statin according to gender, similar benefit for men and women was shown (p value for interaction = 0.127).

This study is not without limitations. Because specific causes of death for each patient were not uniformly available at all sites, this does not include the cause of death and was based on the all-cause mortality not cardiovascular death. Although coronary heart disease remains the most common cause of death in developed countries, it is not possible to enucleate the proportion of deaths which are originated from cardiovascular cause. However, this endpoint is not affected by bias from misclassification of cause of death. And risk factors such as hypertension, diabetes mellitus, dyslipidemia and smoking can contribute to all-cause mortality via additional non-cardiovascular mechanisms. Therefore, the primary endpoint of this study can be an appropriate endpoint to follow. Information regarding statin use only was available for the baseline time point. Analysis according to the treatment patterns of statin can be useful to understand the mitigation of cardiac events. However, data were lacking with regards to statin type and dosage, treatment indication, and duration of therapy. Further, changes in statin medication use that may have occurred beyond baseline CCTA were unavailable. However, that post-test initiation of statins in patients with newly discovered atherosclerosis would likely have resulted in a greater attenuation in risk and a reduction in differences. It is further doubtful that patients on statins at baseline with had evidence of atherosclerotic plaque by CCTA would have had their treatment stopped. Finally, there was the lack of follow-up CCTA to evaluate the progression (or regression) of atherosclerotic burden or change of plaque composition based on statin therapy. In a previous publication, statin use was associated with an increased prevalence and burden of coronary plaques possessing calcium [28]. However, the serial association of plaque composition type to the longitudinal effect of statin was not also addressed. Repeat CCTA for such reasons is not clinically recommended at this point. Although the observational design of the study renders our findings hypothesis-generating, the large multinational cohort increases the generalizability of these findings and augments the paucity of available literature on this topic. Additional large-scale randomized controlled trials designed to evaluate the impact of early statin therapy on outcomes in patients with non-obstructive CAD by CCTA appear warranted. Also, fractional flow reserve (FFR) CT can be useful to overcome the limitation of CCTA to detect coronary stenosis. Current CT has decreased accuracy in the setting of significant calcification and can’t evaluate lesion-specific ischemia. Therefore, FFR CT-based temporal change of anatomy and physiology can be helpful to clarify the effect of statin in CAD.

In conclusion, in this prospective multinational study of patients presenting with anatomically non-obstructive CAD, a heightened risk of adverse cardiac events including all-cause mortality was observed with increasing CAC and SIS among patients not on statin therapy at baseline. This stepwise risk was attenuated among patients on baseline statin therapy after adjustment for clinical covariates. Although we await further confirmation, the current findings are promising in that statin therapy may be associated with mitigating the risk of adverse cardiac events among non-obstructive CAD patients, with no particular threshold of atherosclerotic disease burden.

## Supporting information

S1 File The CONFIRM Registry Charter. This shows the rationale and design of study.
(DOCX)

S2 File TREND checklist. (PDF)
