# Supplementary material for: Usefulness of baseline statin therapy in non-obstructive coronary artery disease by coronary computed tomographic angiography: From the CONFIRM (COronary CT Angiography EvaluatioN For Clinical Outcomes: An InteRnational Multicenter) study
Source: PLoS One. 2018 Dec 12;13(12):e0207194. doi: 10.1371/journal.pone.0207194 (PMC6291090; doi:10.1371/journal.pone.0207194)
Supplement: S1 File — This shows the rationale and design of study. (DOCX) [file pone.0207194.s001.docx]

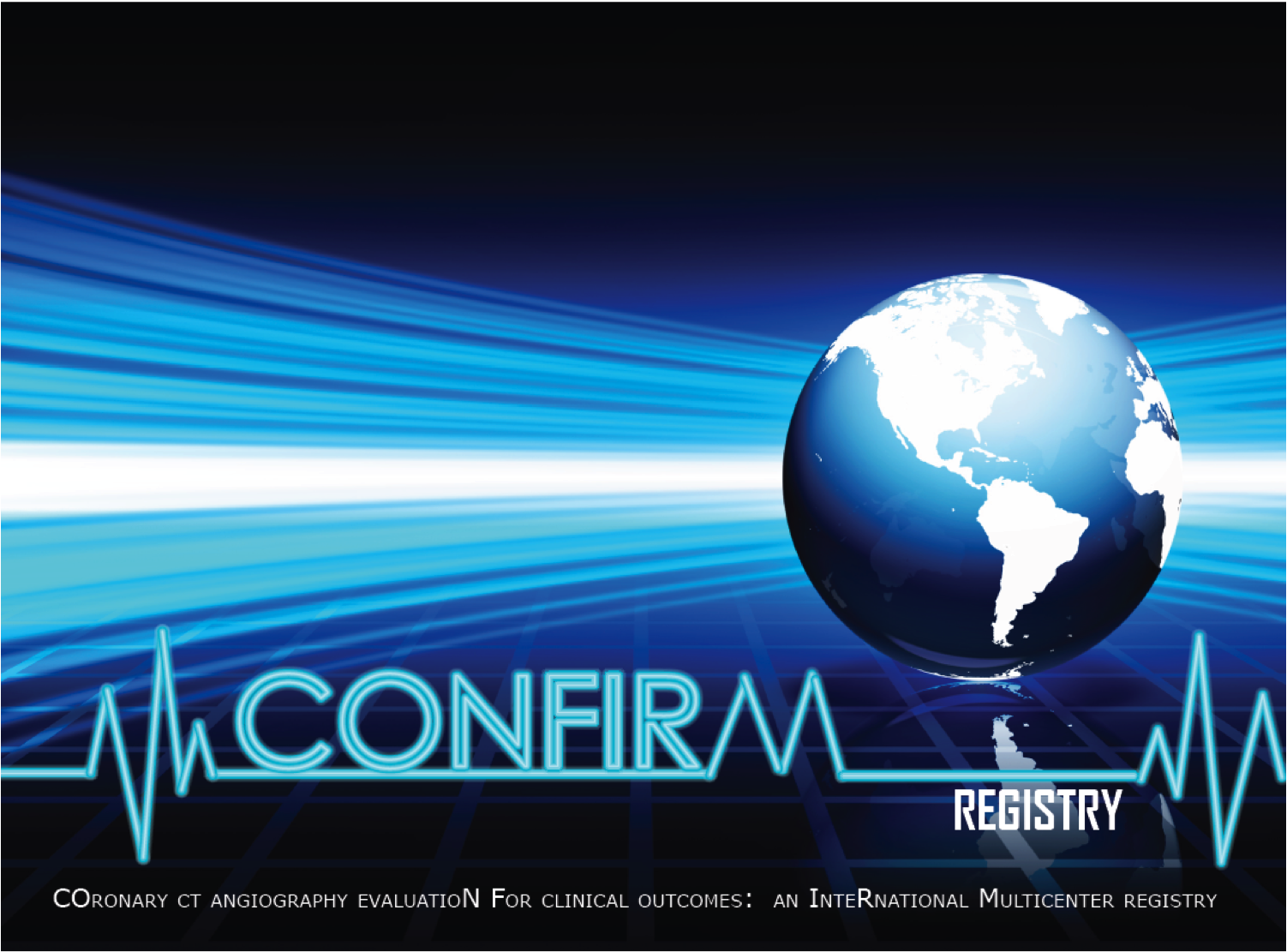


**The CONFIRM Registry Charter**

Table of Contents

1. Study Investigators1

2. Background4

3. Study Objectives1

3.1. Primary Objective2

3.2. Secondary Objectives2

3.3. Other Objectives2

4. Eligibility4

4.1. Patient Eligibility5

4.2. Site Eligibility5

5. Study Organization4

5.1. Collaborative Agreements5

5.2. Data Use Agreements5

5.3. Decision Making5

6. Data Management4

6.1. Data Definitions5

6.2. Data Dictionary5

6.3. Local Site Data Management5

6.4. Data Coding Committee3

6.5. Database Lock5

6.6. Statistical Analysis5

7. Collaboration4

7.1. Project Leads5

7.2. Transparency3

8. Image Storage and Analysis4

8.1. Imaging Repository5

8.2. Imaging Core Laboratories5

9. Study Expansion4

9.1. Additional Patients from Pre-existing Sites5

9.2. Additional Sites/ Investigators5

9.3. Longer-term Follow-up From Pre-existing Sites5

9.4. Transparency3

10. Funding4

11. References4

12. Appendices4

12.1. Data elements5

12.2. Disclosures5

**Study Investigators**

1. **INVESTIGATORS**

**Imaging Core Laboratories:** Matthew J. Budoff MD

Stephan Achenbach MD

**Data Coordinating Center:** James K. Min MD and Allison M. Dunning MS

520 E. 70^th^ Street

Starr Pavilion, ST-433

Weill Cornell Medical College

The New York Presbyterian Hospital

New York, NY 10021

**Contact Information:** Millie Gomez MD

520 E. 70^th^ Street

Starr Pavilion, K415

Weill Cornell Medical College

The New York Presbyterian Hospital

New York, NY 10021

**2. BACKGROUND**

Coronary computed tomographic angiography (CCTA) of 64-detector rows or greater has been recently introduced as a novel non-invasive anatomic method for evaluation of CAD, demonstrating high diagnostic accuracy for detection and exclusion of obstructive CAD(1-3). Several recent reports have also examined the prognostic value of CCTA findings for prediction of future adverse CAD events, but have been generally limited to single centers and by small patient cohorts(4-14). ­­Further, prior studies examining risk stratification by CCTA findings have been primarily restricted to measures of obstructive CAD by maximal luminal diameter stenosis severity on a per-patient or per-vessel basis, with other plaque characteristics visualized by CCTA—including location, distribution, extent, and composition—and non-coronary cardiac findings—including left ventricular systolic function, volume, and regional wall motion—largely neglected within development of prognostic models.

­ To date, no large multicenter study has examined the totality of coronary plaque characteristics and non-coronary cardiac findings as detected by CCTA for optimal refinement of prognostic risk stratification. In this regard, CONFIRM (COronary CT Angiography EvaluatioN For Clinical Outcomes: An InteRnational Multicenter Registry) was developed to provide a vehicle by which CCTA findings could be identified to optimize risk assessment. This report describes the rationale and design features of the CONFIRM registry.

**3. STUDY OBJECTIVES.**

**3.1. OVERALL Objective.** CONFIRM is a prospective, open-label, international, multicenter observational registry designed to evaluate associations between CCTA imaging findings and clinical presentation (cross-sectional) and their ability to predict mortality and major adverse cardiac events (longitudinal) in patients with chronic CAD.

**3.2. Primary Objective.** The primary objective of the CONFIRM registry is to identify, quantify and integrate coronary and non-coronary cardiac findings by CCTA with demographic and clinical data for refinement of risk stratification of individuals with suspected or known CAD.

**3.3. Secondary Objectives.** The secondary objectives of the CONFIRM registry include the following:

1. to determine optimal methods of scoring CCTA to allow for the most accurate risk stratification;
2. to examine prognostic risk assessment­ of CCTA coronary and non-coronary cardiac findings in specific subgroups including but not limited to diabetics, women, and different ethnic groups;
3. to determine the incremental value of CCTA findings beyond coronary artery calcium score (CACS) for prediction of incident adverse CAD events;
4. to associate clinical symptoms and demographic data to CCTA coronary artery findings with an aim at refining estimates of likelihood of CAD for symptomatic individuals with suspected CAD;
5. to associate CCTA coronary and non-coronary cardiac findings with clinical risk factors and symptoms for development of a CCTA global risk score for symptomatic individuals with suspected CAD;
6. to examine post-CCTA resource utilization, particularly as related to rates of downstream invasive coronary angiography;
7. to identify specific coronary artery plaque characteristics identified on CCTA that are associated with ‘culprit’ lesions at the time of incident presentation of acute myocardial infarction.

**3.4. Other Objectives.** Other objectives are anticipated during the evolution of the CONFIRM registry and will be defined in accordance to policies defined in section 5.3.

**4. ELIGIBILITY**

**4.1. Patient Eligibility**. All consecutive patients at cluster sites meeting all inclusion criteria undergoing CCTA of 64-detector rows or greater will be included within the CONFIRM registry. Patient inclusion criteria include:

1. Age > 18 years;
2. evaluation by CCTA with 64-detector rows or greater for CAD evaluation;
3. interpretable CCTA;
4. prospective data collection for CAD risk factors.

No explicit patient exclusion criteria were defined.

**4.2. Site Eligibility.** Each participating site contributing patient-level data to the CONFIRM registry should meet the following site requirements:

1. greater than 200 patients per annum undergoing CCTA by 64-detector rows or greater;
2. incorporation of CCTA into daily clinical practice by members of the medical center other than those involved with the CCTA performance and interpretation; and
3. director of laboratory possessing level III expertise in CCTA.

**5. STUDY ORGANIZATION**

**5.1. Collaborative Agreements.**

In lieu of formal written collaborative agreements, the CONFIRM investigators will agree in writing to adhere to this present CONFIRM registry charter for rules governing action and reporting.

**5.2. Data Use Agreements.**

Data use agreements will be employed only for CONFIRM investigative sites that require them. Data use agreements, when employed, will cover standards of performance; compliance with laws; terms and termination. Data use agreements will also address issues relating to privacy of protected health information, including unauthorized use or disclosure and information regarding safeguards. In no case will indemnity be available for any CONFIRM data use agreement.

**5.3. Decision Making**

In order to facilitate an expedient yet egalitarian decision making process, an executive steering committee (ESC) will be formed. The ESC will consist of 3 members who will perform the following functions:

1. Determine the scientific validity of proposed study topics as submitted by CONFIRM investigators
2. Ensure that excess overlap between proposed study topics submitted by different CONFIRM investigators
3. With the assistance of the other CONFIRM investigators, will devise publication strategies for study topics

If a study topic is proposed by a member of the CONFIRM ESC, that member will recuse himself/herself and the functions of the ESC will be carried out by the other two members of the ESC. In this scenario, if the other two ESC members do not agree upon the scientific validity, degree of overlap or publication strategy, the questions at hand will be brought to the entire CONFIRM investigative team and a vote will be taken with a majority rule governing the path forward.

**6. Data Management**

**6.1. Outcome Definitions**

The following definitions will be used:

Cardiac death will be defined in a manner identical to prior multicenter trials, and will include death due to any of the following: 1) myocardial infarction; 2) heart failure (includes death due to CHF, cardiogenic shock or pulmonary edema. All deaths from hypotension (systolic BP <80mmHg) and/or respiratory failure without other clear etiology will be classified as heart failure); 3) cardiac perforation/pericardial tamponade (all cases of pericardial tamponade will be attributed to cardiac perforation unless other clear etiology identified, e.g., malignancy); 4) arrhythmic death; 5) cerebrovascular accident if related to randomized procedure; 6) unclear cause of death, but cardiac cause cannot be excluded; 7) death due to non-cardiac complication of the procedure (if the procedure is a cardiac procedure), including bleeding, vascular repair, transfusion reaction or bypass surgery.

*Non-cardiac death* will be deaths due to any non-cardiac cause.

*Myocardial Infarction* will be defined in accordance to the universal definition of MI as established by the European Society of Cardiology, ACC, AHA and World Heart Federation.

*CAD-related hospitalization* will be defined as hospitalization for signs or symptoms of unstable angina. Unstable angina will be defined by 1) rest angina, 2) new-onset angina (<2 months), or 3) increasing angina (in intensity, duration and/or frequency. Patients hospitalized for UA will be considered as having experienced CAD-related hospitalization, whether target vessel revascularization is performed or not.

*Target vessel revascularization.* *Late TVR*, which will be defined as TVR occurring ≥90 days following the index diagnostic test (either MPS or CCTA).

*Stroke* will be defined as a neurological deficit lasting ≥24 hours or lasting <24 hours with a brain imaging study showing infarction.

*Congestive heart failure* will be defined by the Framingham Heart Study criteria, and will be made when 2 major criteria are present or 1 major and 2 minor criteria are present concurrently.

*Effective biological radiation dose* will be reported in millisieverts, and will consider the radiation dose administered and absorbed, and the differential biological effects in accordance to type of tissue being irradiated. The effective biological radiation dose will be the sum of equivalent doses of the various types of irradiated tissue, properly weighted for its sensitivity to radiation.

**6.2. Data Dictionary**

A data dictionary will be employed for the merged CONFIRM database, as is delineated in Section 12.1.

**6.3. Local Site Data Management**

CONFIRM investigative sites are required to maintain an electronic database for submission to the Data Coordinating Center. Information relating to patients undergoing CCTA may be recorded on paper case report forms (CRFs), but should be entered into an excel database prior to submission to the DCC. Prior to submission to the DCC, local investigative CONFIRM sites will check data for possible errors, omissions or out-of-range values.

CCTA image files will be stored locally by CONFIRM investigative sites in DICOM format for a minimum of 5 y ears.

**6.4. Data Coding Committee**

The data coding committee will consistent of a biostatistician at the DCC as well as an independent biostatistician not directly associated with the DCC. The data coding committee will function in the following manner: The statistician at the DCC will receive data submissions from CONFIRM investigative sites. Upon receipt, the DCC performed an additional check for possible errors, omissions or out-of-range values. After cleaning by a dedicated biostatistician at WCMC, the CONFIRM dataset was sent to an independent biostatistician for checks of database cleanliness. After agreement by both the DCC and independent statistician that the database is ready for analysis, the CONFIRM database will be considered ready for database lock.

**6.5. Database Lock**

Database lock will occur after agreement of readiness by the data coding committee, the ESC and the entire CONFIRM investigative group. Databases will be locked for a minimum of 18 months, but can be reopened at the behest of the ESC to enable longer follow-up of patients within the CONFIRM registry, for addition of patients undergoing CCTA at CONFIRM investigative sites, and for addition of patients undergoing CCTA at sites being considered for addition into the CONFIRM registry.

**6.6. Statistical Analysis**

Descriptive, univariate and multivariate analyses will be conducted. For the primary objective, the proportional hazards assumptions will be tested for each CCTA finding in relationship to mortality, and a multivariate Cox proportional hazards model will be used to evaluate the association of CCTA findings with the risk of death from all causes. Regression models will be adjusted for demographic and clinical variables and symptoms, and likelihood ratio tests will be used to determine a multivariable P value. Backward stepwise regression will be used to select an independently predictive set of CCTA findings. Receiver operating curves will be plotted for models with clinical data alone and in conjunction with CCTA findings, and a c-statistic will be calculated to determine the discriminatory ability of CCTA findings. In addition to the secondary analyses described above, CONFIRM investigators are also encouraged to propose analyses with pre-specification of hypotheses and statistical plans. To address multiple hypothesis testing, statistical significance will account for false discovery rates.

CONFIRM investigative sites will perform statistical analyses using local site statisticians or else will submit statistical analysis plans to the DCC for performance. In the former case, the DCC will confirm findings by replication of analyses; while in the latter case, the DCC findings will be confirmed by an independent 3^rd^ party biostatistician.

Given the multiple sites in CONFIRM, an important consideration for analysis is heterogeneity of data. Analyses with inclusion of exposures, outcomes or covariates with significant heterogeneity will be performed with a random effects model by site, and confirmed with sensitivity analysis removing each site sequentially from analysis to estimate the standard error of the mean.

Patients with incomplete CCTA or outcome reporting are excluded from CONFIRM analyses, as are patients with missing age and gender. For patients with other missing-at-random covariate values, multiple imputation procedures will be performed by site. Patients with missing demographics such as age and gender will be excluded.

For sites that did not collect one entire category of risk factors such as family history, a dummy value for “missing by site” will be assigned as a covariate. Sites that did not collect more than one entire category of risk factors will be excluded from the CONFIRM data set. For each primary analysis, sensitivity analysis will be performed with Monte Carlo simulations of 100 imputations to estimate the standard error of the mean.

**7. COLLABORATION**

**7.1. Project Leads**

For each study topic approved by the ESC, there will be a project leader(s). A maximum of two project leaders will be assigned for each study topic. Projects are encouraged to have two project leaders – from separate CONFIRM investigative sites – in order to promote collaboration between CONFIRM sites. If only one CONFIRM site is leading the study project, there will be only one project leaders.

**7.2. Transparency**

Due to the large number of study sites, transparency and openness of communication is of high importance. A “wiki” site will be formed where site documents, analyses, abstracts and manuscripts can be viewed. Each CONFIRM investigator will be given a unique password to ensure that only appropriate parties can access the data. For this site, administrator privileges have been developed in addition to mechanisms by which any changes to documents can be linked to the investigator who changed them.

In addition, communication between individual parties within CONFIRM is discouraged, in lieu of the preferred method of communicating with all parties simultaneously.

CONFIRM investigators who suspect impropriety by other CONFIRM investigators are encouraged to discuss such issues with the ESC. If one of the investigators suspected of impropriety is a member of the ESC, that investigator is encouraged to discuss issues with the other two members of the ESC. If no adequate resolution results from the discussions with the ESC, investigators are encouraged to raise concerns with the entire CONFIRM investigative group for discussion.

To enable continued open discussion regarding status of study topics, a conference call will be planned for approximately 1 time per month.

**8. IMAGE STORAGE AND ANALYSIS**

**8.1. Imaging Repository**

No central imaging repository is required for all CONFIRM CCTA scans. For study projects that require dedicated image re-analysis, images can be sent to the Data Coordinating Center. The DCC will be responsible for sending the images to the Imaging Core Laboratories. Images sent from the local sites to the DCC should be de-identified with labels that correspond to the patient identification numbers as submitted with the CONFIRM data. The DCC will retain these de-identified patient identification numbers when sending to the imaging core laboratories

At the investigative site’s convenience and if agreeable by the CONFIRM ESC, images may also be directly sent to the Imaging Core Laboratories.

**8.2. Imaging Core Laboratories**

The ESC will designate which laboratories will serve as the Imaging Core Laboratories. For all studies requiring imaging re-analysis, a minimum of 1 Imaging Core Laboratory will be chosen, with two Imaging Core Laboratories preferred. Whether one or two Imaging Core Laboratories are used, interobserver and intraobserver and (in the case two laboratories are used) inter-laboratory variability should be measured.

**9. STUDY EXPANSION**

**9.1. Additional Patients from Pre-existing Sites**

For expansion of the CONFIRM database, additional patients undergoing CCTA from pre-existing CONFIRM investigative sites will be considered for addition to the CONFIRM database. These additions will occur at defined periods, as decided by the ESC. It is anticipated that a minimum of 18 months after database lock should occur to allow for meaningful addition of patients.

**9.2. Additional Sites/ Investigators**

The ESC, in collaboration with the CONFIRM investigators, will decide whether additional sites should be added. Given the large number of patients already in the CONFIRM registry, the addition of sites should be additive towards patient subsets that are not well-represented in CONFIRM already. The addition of these sites will be done at the time of database ‘unlocking,’ in order to assure that all CONFIRM investigators have access to a uniform dataset.

**9.3. Longer-term Follow-up from Pre-existing Sites**

At the time of database ‘unlocking,’ CONFIRM sites are encouraged to add to the present database by providing longer-term follow-up. The ascertainment of this follow-up should occur in a manner similar to the original data contribution.

**9.4. Transparency**

**10. FUNDING**

Funding applications to non-profit or for-profit entities will encouraged. Prior to the submission of any application for funding, any individual CONFIRM investigator will be required to obtain approval from the CONFIRM ESC.

Successful applications from individual CONFIRM investigative sites that receive funding will deposit the funding at the respective individual institutions. No central funding repository will exist for CONFIRM. Open accounting with regular updates on funding spent and remaining will be expected during the CONFIRM conference calls. In anticipation of calls, investigative sites with funding should send out a .pdf or excel document with accounting for CONFIRM investigator review.

**11. REFERENCES**

1. Budoff MJ, Dowe D, Jollis JG, et al. Diagnostic performance of 64-multidetector row coronary computed tomographic angiography for evaluation of coronary artery stenosis in individuals without known coronary artery disease: results from the prospective multicenter ACCURACY (Assessment by Coronary Computed Tomographic Angiography of Individuals Undergoing Invasive Coronary Angiography) trial. J Am Coll Cardiol 2008; 52:1724-32.

2. Meijboom WB, Meijs MF, Schuijf JD, et al. Diagnostic accuracy of 64-slice computed tomography coronary angiography: a prospective, multicenter, multivendor study. J Am Coll Cardiol 2008; 52:2135-44.

3. Miller JM, Rochitte CE, Dewey M, et al. Diagnostic performance of coronary angiography by 64-row CT. N Engl J Med 2008; 359:2324-36.

4. Min JK, Shaw LJ, Devereux RB, et al. Prognostic value of multidetector coronary computed tomographic angiography for prediction of all-cause mortality. J Am Coll Cardiol 2007; 50:1161-70.

5. Min JK, Feignoux J, Treutenaere J, Laperche T, Sablayrolles J. The prognostic value of multidetector coronary CT angiography for the prediction of major adverse cardiovascular events: a multicenter observational cohort study. Int J Cardiovasc Imaging 2010; 26:721-8.

6. Min JK, Lin FY, Dunning AM, et al. Incremental prognostic significance of left ventricular dysfunction to coronary artery disease detection by 64-detector row coronary computed tomographic angiography for the prediction of all-cause mortality: results from a two-centre study of 5330 patients. Eur Heart J 2010; 31:1212-9.

7. Chow BJ, Wells GA, Chen L, et al. Prognostic value of 64-slice cardiac computed tomography severity of coronary artery disease, coronary atherosclerosis, and left ventricular ejection fraction. J Am Coll Cardiol 2010; 55:1017-28.

8. Hadamitzky M, Meyer T, Hein F, et al. Prognostic value of coronary computed tomographic angiography in asymptomatic patients. Am J Cardiol 2010; 105:1746-51.

9. Hadamitzky M, Hein F, Meyer T, et al. Prognostic value of coronary computed tomographic angiography in diabetic patients without known coronary artery disease. Diabetes Care 2010; 33:1358-63.

10. Hadamitzky M, Freissmuth B, Meyer T, et al. Prognostic value of coronary computed tomographic angiography for prediction of cardiac events in patients with suspected coronary artery disease. JACC Cardiovasc Imaging 2009; 2:404-11.

11. Ostrom MP, Gopal A, Ahmadi N, et al. Mortality incidence and the severity of coronary atherosclerosis assessed by computed tomography angiography. J Am Coll Cardiol 2008; 52:1335-43.

12. van Werkhoven JM, Schuijf JD, Gaemperli O, et al. Incremental prognostic value of multi-slice computed tomography coronary angiography over coronary artery calcium scoring in patients with suspected coronary artery disease. Eur Heart J 2009; 30:2622-9.

13. van Werkhoven JM, Schuijf JD, Gaemperli O, et al. Prognostic value of multislice computed tomography and gated single-photon emission computed tomography in patients with suspected coronary artery disease. J Am Coll Cardiol 2009; 53:623-32.

14. van Werkhoven JM, Cademartiri F, Seitun S, et al. Diabetes: prognostic value of CT coronary angiography--comparison with a nondiabetic population. Radiology 2010; 256:83-92.

**12. APPENDICES**

**12.1. Data elements**

The following data elements are encouraged for acquisition by CONFIRM sites:

| **Variable name** | **Significance** | **Values** |
| --- | --- | --- |
| **Registry-specific** |  |  |
| **Key** | Unique registry ID | Consecutive integers |
| **Site** | Site location | 1= THVI, 2=Albany, 3=CSMC, 4=Munich, 5=Ontario, 6=Harbor, 7=Ehrlangen, 8=WHH |
| **Site Location** | Site Location text |  |
| **Indication, free text** | Free text | 1=Chest symptoms (pain, tightness, pressure, palpitations);2= Dyspnea; 3= Asymptomatic; 4=Preoperative; 5=EP; 6=Congenital; 7=Other |
| **ICD-9 code** | ICD-9 code | ICD-9 code |
| **Location** | Outpatient vs. ER vs. Inpatient | 1=Outpatient; 2=ER; 3=Inpatient |
| **Demographics** |  |  |
| **Date of study** | **Date of Study** | **Date format** |
| **DOB** | Date of birth | Date format |
| **Age** | Calculated age at time of scan | Age in years, integer |
| **Sex** | Sex | 1 = Male, 0 = Female |
| **BMI** | BMI | Value |
| **Ethnicity** | Patient self-reported ethnicity | 1 = Caucasian, 2 = African, 3 = Latin American, 4 = East asian, 5 = South Asian, 6 = Middle eastern, 7 = Other or mixed |
| **Height** | Self reported height | Height in inches or cm (please specify) |
| **Weight** | Self reported weight | Weight in pounds or kg (please specify) |
| **Past_MI** | Self-reported past MI | 1= yes, 0 = no, -1 = unknown |
| **Past PCI** | Self-reported past PCI | 1=yes, 0=no, -1=unknown |
| **Past CABG** | Self-reported past CABG | 1=yes, 0=no, -1=unknown |
| **Past stress test** | Self-reported past stress test | 1=yes, 0=no, -1=unknown |
| **HTN** | Self-reported HTN or med | 1=yes, 0=no, -1=unknown |
| **DM** | Self-reported DM or med | 1=yes, 0=no, -1=unknown |
| **Dyslipidemia (CHOL)** | Self-reported dyslipidemia or med | 1=yes, 0=no, -1=unknown |
| **Date of Lipid Panel** | Date | Date format: 06/01/09 |
| **Total cholesterol** | Laboratory value | Value (mg/dl) |
| **LDL cholesterol** | Laboratory value | Value (mg/dl) |
| **HDL cholesterol** | Laboratory value | Value (mg/dl) |
| **FAMHX** | Self-reported family history (age<55 M, 65 F) | 1=yes, 0=no, -1=unknown |
| **SMOKEcurrent** | Self-reported current smoking | 1=yes, 0=no, -1=unknown |
| **SMOKEpast** | Self-reported past smoking | 1=yes, 0=no, -1=unknown |
| **Renal insufficiency** | Self-reported renal insufficiency | 1=yes, 0=no, -1=unknown |
| **Date of Serum Creatinine** | Date | Date format: 06/01/09 |
| **Creatinine** | Laboratory value | Value (mg/dl) |
| **Peripheral vascular disease (PV_Disease)** | Self-reported peripheral arterial disease | 1=yes, 0=no, -1=unknown |
| **Cerebrovascular disease (CV_Disease)** | Self-reported cerebrovascular disease | 1=yes, 0=no, -1=unknown |
| **Symptoms** |  |  |
| **Frequency of Chest Pain** | Episodes/week | 0=0, 1=1-2x/week, 2=3-5/week, 4=>5x/week, -1=unknown |
| **Chest pain** | Any chest pain or tightness? | 1=yes, 0=no, -1=unknown |
| **CP worse with exertion (Exertion)** | Chest pain or tightness worse with exertion | 1=yes, 0=no, -1=unknown |
| **Relief_with_minutes (Relief)** | Relief with rest or NTG? | 1=yes, 0=no, -1=unknown |
| **Typicality of chest pain** | Chest pain typicality | 0 = no pain, 1 = noncardiac, 2 = atypical, 3 = typical |
| **Shortness of breath (SOB)** | Dyspnea? | 1=yes, 0=no, -1=unknown |
| **Suspected ACS (ACS)** | Suspected ACS? | 1=yes, 0=no, -1=unknown |
| **Clinical Events** |  |  |
| **DOD** | Date of death | Date format: 06/01/09 |
| **date_PCI** | Date of PCI | Date format: 06/01/09 |
| **date_CABG** | Date of CABG | Date format: 06/01/09 |
| **date_cath** | Date of cath | Date format: 06/01/09 |
| **date_ACS** | Date of ACS | Date format: 06/01/09 |
| **date_MI** | Date of MI (if type not specified) | Date format: 06/01/09 |
| **date_STEMI** | Date of STEMI | Date format: 06/01/09 |
| **date_NSTEMI** | Date of NSTEMI | Date format: 06/01/09 |
| **date_cardiac_hosp** | Date of hospitalization | **Date format: 06/01/09** |
| **date_coronary_hosp** | Date of hospitalization | **Date format: 06/01/09** |
| **Coronary CT Scan Variables** |  |  |
| **Native coronary arteries** |  |  |
| **Coronary artery calcium score (CalciumScore)** | Agatston units | Value (Agatston units) |
| **LM_severity** | LM severity | 0 = none, 1 = 1-24%, 2=25-49%, 3=1-49%, 4=50-69%, 5=70-99%, 6=100%, 7=nonevaluable |
| **LM_composition** | LM composition | 1 = noncalcified, 2=mixed (30-70% calcified/noncalcified), 3=calcified |
| **pLAD_severity** | pLAD severity | 0 = none, 1 = 1-24%, 2=25-49%, 3=1-49%, 4=50-69%, 5=70-99%, 6=100%, 7=nonevaluable |
| **pLAD_composition** | pLAD composition | 1 = noncalcified, 2=mixed (30-70% calcified/noncalcified), 3=calcified |
| **mLAD_severity** | mLAD severity | 0 = none, 1 = 1-24%, 2=25-49%, 3=1-49%, 4=50-69%, 5=70-99%, 6=100%, 7=nonevaluable |
| **mLAD_composition** | mLAD composition | 1 = noncalcified, 2=mixed (30-70% calcified/noncalcified), 3=calcified |
| **dLAD_severity** | dLAD severity | 0 = none, 1 = 1-24%, 2=25-49%, 3=1-49%, 4=50-69%, 5=70-99%, 6=100%, 7=nonevaluable |
| **dLAD_composition** | dLAD composition | 1 = noncalcified, 2=mixed (30-70% calcified/noncalcified), 3=calcified |
| **DIAG1_severity** | Diagonal 1 severity | 0 = none, 1 = 1-24%, 2=25-49%, 3=1-49%, 4=50-69%, 5=70-99%, 6=100%, 7=nonevaluable |
| **DIAG1_composition** | Diagonal 1 composition | 1 = noncalcified, 2=mixed (30-70% calcified/noncalcified), 3=calcified |
| **DIAG2_severity** | Diagonal 2 severity | 0 = none, 1 = 1-24%, 2=25-49%, 3=1-49%, 4=50-69%, 5=70-99%, 6=100%, 7=nonevaluable |
| **DIAG2_composition** | Diagonal 2 composition | 1 = noncalcified, 2=mixed (30-70% calcified/noncalcified), 3=calcified |
| **pLCX_severity** | pLCX severity | 0 = none, 1 = 1-24%, 2=25-49%, 3=1-49%, 4=50-69%, 5=70-99%, 6=100%, 7=nonevaluable |
| **pLCX_composition** | pLCX composition | 1 = noncalcified, 2=mixed (30-70% calcified/noncalcified), 3=calcified |
| **dLCX_severity** | dLCX severity | 0 = none, 1 = 1-24%, 2=25-49%, 3=1-49%, 4=50-69%, 5=70-99%, 6=100%, 7=nonevaluable |
| **dLCX_composition** | dLCX composition | 1 = noncalcified, 2=mixed (30-70% calcified/noncalcified), 3=calcified |
| **OM1_severity** | OM 1 severity | 0 = none, 1 = 1-24%, 2=25-49%, 3=1-49%, 4=50-69%, 5=70-99%, 6=100%, 7=nonevaluable |
| **OM1_composition** | OM 1 composition | 1 = noncalcified, 2=mixed (30-70% calcified/noncalcified), 3=calcified |
| **OM2_severity** | OM 2 severity | 0 = none, 1 = 1-24%, 2=25-49%, 3=1-49%, 4=50-69%, 5=70-99%, 6=100%, 7=nonevaluable |
| **OM2_composition** | OM 2 composition | 1 = noncalcified, 2=mixed (30-70% calcified/noncalcified), 3=calcified |
| **LeftPL_severity** | LeftPL severity | 0 = none, 1 = 1-24%, 2=25-49%, 3=1-49%, 4=50-69%, 5=70-99%, 6=100%, 7=nonevaluable |
| **LeftPL_composition** | LeftPL composition | 1 = noncalcified, 2=mixed (30-70% calcified/noncalcified), 3=calcified |
| **pRCA_severity** | pRCA severity | 0 = none, 1 = 1-24%, 2=25-49%, 3=1-49%, 4=50-69%, 5=70-99%, 6=100%, 7=nonevaluable |
| **pRCA_composition** | pRCA composition | 1 = noncalcified, 2=mixed (30-70% calcified/noncalcified), 3=calcified |
| **mRCA_severity** | mRCA severity | 0 = none, 1 = 1-24%, 2=25-49%, 3=1-49%, 4=50-69%, 5=70-99%, 6=100%, 7=nonevaluable |
| **mRCA_composition** | mRCA composition | 1 = noncalcified, 2=mixed (30-70% calcified/noncalcified), 3=calcified |
| **dRCA_severity** | dRCA severity | 0 = none, 1 = 1-24%, 2=25-49%, 3=1-49%, 4=50-69%, 5=70-99%, 6=100%, 7=nonevaluable |
| **dRCA_composition** | dRCA composition | 1 = noncalcified, 2=mixed (30-70% calcified/noncalcified), 3=calcified |
| **PDA_severity** | PDA severity | 0 = none, 1 = 1-24%, 2=25-49%, 3=1-49%, 4=50-69%, 5=70-99%, 6=100%, 7=nonevaluable |
| **PDA_composition** | PDA composition | 1 = noncalcified, 2=mixed (30-70% calcified/noncalcified), 3=calcified |
| **RightPL_severity** | RightPL severity | 0 = none, 1 = 1-24%, 2=25-49%, 3=1-49%, 4=50-69%, 5=70-99%, 6=100%, 7=nonevaluable |
| **RightPL_composition** | RightPL composition | 1 = noncalcified, 2=mixed (30-70% calcified/noncalcified), 3=calcified |
| **PL_severity** | PL severity (if direction not specified) | 0 = none, 1 = 1-24%, 2=25-49%, 3=1-49%, 4=50-69%, 5=70-99%, 6=100%, 7=nonevaluable |
| **PL_composition** | PL composition (if direction not specified) | 1 = noncalcified, 2=mixed (30-70% calcified/noncalcified), 3=calcified |
| **ANOMALOUS** | Anomalous coronary artery | 0=no, 1=yes |
| **RightDominant** | Right or left dominance | 1 = right dominant, 0 = left dominant |
| **Coronary bypass grafts** |  |  |
| **LIMA** | LIMA destination | 1=LAD, 2=LCX, 3=RCA, 4=Diagonal, 5=OM, 6=PDA, 7=RPL |
| **LIMA_sev** | LIMA severity | 0 = none, 1 = 1-24%, 2=25-49%, 3=1-49%, 4=50-69%, 5=70-99%, 6=100%, 7=nonevaluable |
| **Other arterial graft** | RIMA or radial graft destination | 1=LAD, 2=LCX, 3=RCA, 4=Diagonal, 5=OM, 6=PDA, 7=RPL |
| **Other arterial graft** | RIMA severity | 0 = none, 1 = 1-24%, 2=25-49%, 3=1-49%, 4=50-69%, 5=70-99%, 6=100%, 7=nonevaluable |
| **SVG1** | SVG1 destination | 1=LAD, 2=LCX, 3=RCA, 4=Diagonal, 5=OM, 6=PDA, 7=RPL |
| **SVG1_sev** | SVG1 severity | 0 = none, 1 = 1-24%, 2=25-49%, 3=1-49%, 4=50-69%, 5=70-99%, 6=100%, 7=nonevaluable |
| **SVG2** | SVG2 destination | 1=LAD, 2=LCX, 3=RCA, 4=Diagonal, 5=OM, 6=PDA, 7=RPL |
| **SVG2_sev** | SVG2 severity | 0 = none, 1 = 1-24%, 2=25-49%, 3=1-49%, 4=50-69%, 5=70-99%, 6=100%, 7=nonevaluable |
| **SVG3** | SVG3 destination | 1=LAD, 2=LCX, 3=RCA, 4=Diagonal, 5=OM, 6=PDA, 7=RPL |
| **SVG3_sev** | SVG3 severity | 0 = none, 1 = 1-24%, 2=25-49%, 3=1-49%, 4=50-69%, 5=70-99%, 6=100%, 7=nonevaluable |
| **SVG4** | SVG4 destination | 1=LAD, 2=LCX, 3=RCA, 4=Diagonal, 5=OM, 6=PDA, 7=RPL |
| **SVG4_sev** | SVG4 severity | 0 = none, 1 = 1-24%, 2=25-49%, 3=1-49%, 4=50-69%, 5=70-99%, 6=100%, 7=nonevaluable |
| **LM_stent** | LM stent | 0=no, 1=yes |
| **LM_ISR** | LM in-stent restenosis | 0 = none, 1 = 1-24%, 2=25-49%, 3=1-49%, 4=50-69%, 5=70-99%, 6=100%, 7=nonevaluable |
| **LAD_stent** | LAD stent (if not broken up) | 0=no, 1=yes |
| **LAD_ISR** | LAD in-stent restenosis (if not broken up) | 0 = none, 1 = 1-24%, 2=25-49%, 3=1-49%, 4=50-69%, 5=70-99%, 6=100%, 7=nonevaluable |
| **pLAD_stent** | pLAD stent | 0=no, 1=yes |
| **pLAD_ISR** | pLAD in-stent restenosis | 0 = none, 1 = 1-24%, 2=25-49%, 3=1-49%, 4=50-69%, 5=70-99%, 6=100%, 7=nonevaluable |
| **mLAD_stent** | mLAD stent | 0=no, 1=yes |
| **mLAD_ISR** | mLAD in-stent restenosis | 0 = none, 1 = 1-24%, 2=25-49%, 3=1-49%, 4=50-69%, 5=70-99%, 6=100%, 7=nonevaluable |
| **dLAD_stent** | dLAD stent | 0=no, 1=yes |
| **dLAD_ISR** | dLAD in-stent restenosis | 0 = none, 1 = 1-24%, 2=25-49%, 3=1-49%, 4=50-69%, 5=70-99%, 6=100%, 7=nonevaluable |
| **DIAG1_stent** | Diagonal 1 stent | 0=no, 1=yes |
| **DIAG1_ISR** | Diagonal 1 in-stent restenosis | 0 = none, 1 = 1-24%, 2=25-49%, 3=1-49%, 4=50-69%, 5=70-99%, 6=100%, 7=nonevaluable |
| **DIAG2_stent** | Diagonal 2 stent | 0=no, 1=yes |
| **DIAG2_ISR** | Diagonal 2 in-stent restenosis | 0 = none, 1 = 1-24%, 2=25-49%, 3=1-49%, 4=50-69%, 5=70-99%, 6=100%, 7=nonevaluable |
| **LCX_stent** | LCX stent (if not broken up) | 0=no, 1=yes |
| **LCX_ISR** | LCX in-stent restenosis (if not broken up) | 0 = none, 1 = 1-24%, 2=25-49%, 3=1-49%, 4=50-69%, 5=70-99%, 6=100%, 7=nonevaluable |
| **pLCX_stent** | pLCX stent | 0=no, 1=yes |
| **pLCX_ISR** | pLCX in-stent restenosis | 0 = none, 1 = 1-24%, 2=25-49%, 3=1-49%, 4=50-69%, 5=70-99%, 6=100%, 7=nonevaluable |
| **dLCX_stent** | dLCX stent | 0=no, 1=yes |
| **dLCX_ISR** | dLCX in-stent restenosis | 0 = none, 1 = 1-24%, 2=25-49%, 3=1-49%, 4=50-69%, 5=70-99%, 6=100%, 7=nonevaluable |
| **OM1_stent** | OM 1 stent | 0=no, 1=yes |
| **OM1_ISR** | OM 1 in-stent restenosis | 0 = none, 1 = 1-24%, 2=25-49%, 3=1-49%, 4=50-69%, 5=70-99%, 6=100%, 7=nonevaluable |
| **LeftPL_stent** | LeftPL stent | 0=no, 1=yes |
| **LeftPL_ISR** | LeftPL in-stent restenosis | 0 = none, 1 = 1-24%, 2=25-49%, 3=1-49%, 4=50-69%, 5=70-99%, 6=100%, 7=nonevaluable |
| **RCA_stent** | RCA stent (if not broken up) | 0=no, 1=yes |
| **RCA_ISR** | RCA in-stent restenosis (if not broken up) | 0 = none, 1 = 1-24%, 2=25-49%, 3=1-49%, 4=50-69%, 5=70-99%, 6=100%, 7=nonevaluable |
| **pRCA_stent** | pRCA stent | 0=no, 1=yes |
| **pRCA_ISR** | pRCA in-stent restenosis | 0 = none, 1 = 1-24%, 2=25-49%, 3=1-49%, 4=50-69%, 5=70-99%, 6=100%, 7=nonevaluable |
| **mRCA_stent** | mRCA stent | 0=no, 1=yes |
| **mRCA_ISR** | mRCA in-stent restenosis | 0 = none, 1 = 1-24%, 2=25-49%, 3=1-49%, 4=50-69%, 5=70-99%, 6=100%, 7=nonevaluable |
| **dRCA_stent** | dRCA stent | 0=no, 1=yes |
| **dRCA_ISR** | dRCA in-stent restenosis | 0 = none, 1 = 1-24%, 2=25-49%, 3=1-49%, 4=50-69%, 5=70-99%, 6=100%, 7=nonevaluable |
| **PDA_stent** | PDA stent | 0=no, 1=yes |
| **PDA_ISR** | PDA in-stent restenosis | 0 = none, 1 = 1-24%, 2=25-49%, 3=1-49%, 4=50-69%, 5=70-99%, 6=100%, 7=nonevaluable |
| **RightPL_stent** | RightPL stent | 0=no, 1=yes |
| **RightPL_ISR** | RightPL in-stent restenosis | 0 = none, 1 = 1-24%, 2=25-49%, 3=1-49%, 4=50-69%, 5=70-99%, 6=100%, 7=nonevaluable |
| **PL_stent** | PL stent (if not broken up) | 0=no, 1=yes |
| **PL_ISR** | PL in-stent restenosis (if not broken up) | 0 = none, 1 = 1-24%, 2=25-49%, 3=1-49%, 4=50-69%, 5=70-99%, 6=100%, 7=nonevaluable |
| **Cardiac Non-Coronary CT findings** |  |  |
| **LOW_EF** | EF <50% by visual or quantitative estimate | 0=no, 1=yes |
| **EFinPercent** | Calculated ejection fraction | % |
| **LVEDV** | LVEDV | cc |
| **LVESV** | LVESV | cc |
| **RWMA** | Regional wall motion abnormalities | 0=no, 1=yes |
| **EF by other modality** | Ejection fraction | % |
| **Ascending aortic size** | Ascending aortic size | cm |
| **AO_dissection** | Presence of aortic dissection | 0=no, 1=yes |
| **Congenital_Disease** | Presence of congenital cardiac anomalies | 0=no, 1=yes |
| **CT Scan Parameters** |  |  |
| **Nitroglycerin** | Nitroglycerin | 0=no, 1=yes |
| **Contrast amount** | Amount of contrast | cc |
| **Scan Type** | ECG triggering | 1=Retrospective, 2=Prospective |
| **CT Scan Type** | Type of scanner | 1=64-slice single source, 2=dual source, 3=16-slice, 4=EBCT |
| **mA** | mA for CCTA | mA |
| **kV** | kV for CCTA | kV |
| **DLP, coronary CT angiogram (DLP CCTA)** | Dose length product, CCTA | **(msv mGy^-1^ cm^-1^ )** |
| **DLP, total** | Dose length product, total examination | **(msv mGy^-1^ cm^-1^ )** |
| **Study quality** | Study quality | 1=Excellent, 2=Satisfactory, 3=Poor |
| **Medications** |  |  |
| **ACEI** | ACE Inhibitor | 0=no, 1=yes |
| **ASA** | Aspirin | 0=no, 1=yes |
| **AT2 Antagonist** | Angiotensin 2 Antagonist | 0=no, 1=yes |
| **BB** | Beta blocker | 0=no, 1=yes |
| **Ca Blocker** | Calcium channel blocker | 0=no, 1=yes |
| **Diuretics** | Diuretic | 0=no, 1=yes |
| **Coumadin** | Coumadin | 0=no, 1=yes |
| **Hypoglycemics** | Hypoglycemics | 0=no, 1=yes |
| **Metformin** | Metformin | 0=no, 1=yes |
| **Insulin** | Insulin | 0=no, 1=yes |
| **Lipid lowering agents** | Lipid lowering agents, non-statins | 0=no, 1=yes |
| **Statin** | Statins | 0=no, 1=yes |
| **Nitrates** | Nitrates | 0=no, 1=yes |
| **Clopidogrel** | Clopidogrel | 0=no, 1=yes |
| **Survival Parameters** |  |  |
| **Death** | death | 0=no, 1=yes |
| **Last seen** | Date of Social Security Death Index or last visit | Date format: 06/01/09 |
| **Time Follow Up** | follow up time in days | value (days) |
| **Per Patient/Per Artery Findings** |  |  |
| **Obstructive CAD** | Per Patient Obstructive CAD | 0 = Normal 1 = <50% stenosis 2 = >50% stenosis |
| **LM_50** | Left Main Obstrutive Stenosis | 0 = <50% Stenosis in LM, 1 = >50% Stenosis in LM |
| **LAD_50** | LAD Obstructive Stenosis | 0 = <50% Stenosis in LAD, 1 = >50% Stenosis in LAD |
| **LCX_50** | LCX Obstructive Stenosis | 0 = <50% Stenosis in LCX, 1 = >50% Stenosis in LCX |
| **RCA_50** | RCA Obstructive Stenosis | 0 = <50% Stenosis in RCA, 1 = >50% Stenosis in RCA |

­

**12­.2. Disclosures**
